# A theory for spiral wave drift induced by ac and polarized electric fields in chemical excitable media

**DOI:** 10.1038/s41598-017-09092-6

**Published:** 2017-08-17

**Authors:** Teng-Chao Li, Xiang Gao, Fei-Fei Zheng, De-Bei Pan, Bo Zheng, Hong Zhang

**Affiliations:** 1 Zhejiang Institute of Modern Physics and Department of Physics, Zhejiang University, Hangzhou, 310027 China; 2 School of Physics and Information Technology, Shaanxi Normal University, Xi’an, 710062 China; 3 Max-Planck Institute for Dynamics and Self-Organisation, Gottingen, D-37077 Germany

## Abstract

Spiral waves are shown to undergo directional drifts in the presence of ac and polarized electric fields when their frequencies are twice of the spiral frequencies. Here, we propose a quantitative description for the spiral wave drift induced by weak electric fields, and provide the explicit equations for the spiral wave drift speed and direction. Numerical simulations are performed to demonstrate the quantitative agreement with analytical results in both weakly and highly excitable media.

## Introduction

Excitable media represent a wide class of nonequilibrium systems which play an important role in physical, chemical, and biological applications^1, 2^. Spiral waves are among the most paradigmatic examples of spatiotemporal self-organizing structures in excitable media, such as cardiac tissue^3, 4^, aggregations of Dictyostelium discoideum amoebae^5^, the oxidation of CO on platinum^6^, and the Belousov-Zhabotinsky (BZ) reaction^7^. In cardiology, such self-sustained spiral wave activities play an essential role in cardiac arrhythmia and fibrillation^8–13^. This, with the intrinsic interest of those structures, has led to an important research effort in order to understand the dynamic of spiral waves.

The BZ reaction is the most extensively studied system which supports spiral waves. The authors in ref. 14 reported the first experimental observation of a directional drift of the spiral wave along a straight line, during the periodic modulation of the excitability of an excitable medium with a frequency close to the nature rotation frequency of the spiral. This directional drift phenomenon for spiral waves subjected to the external periodic forcing has been confirmed in numerous experiments as well as numerical simulations. And its mechanism has also been studied in refs. 15–28. Based on the case of a weakly excitable medium, an initial mathematical treatment of the directional drift for rigidly rotating spiral waves under a periodic forcing was provided^23^. This approach is based on a kinematic model of spiral dynamics in which one disregards the thickness of the excited area, and models the spiral as a one-dimensional curve. In ref. 24, the periodic forcing of spiral waves was considered from a dynamically systematic view point. It was shown that much of the spiral behavior could be deduced simply from the interaction of dynamics with system symmetries. In ref. 25, an asymptotic derivation of a kinematic theory for the spiral wave motion in the weakly excitable and free-boundary limit was presented. The mechanism of the directional drift of spiral waves subjected to the external periodic forcing was studied and a drift velocity formula for spirals was obtained. In refs. 26–28, the directional drift for rigidly rotating spiral waves under the periodic forcing was studied by using the response function theory for both weak and high excitabilities.

In the presence of a dc electric field, spiral waves in the BZ reaction drift with a velocity whose two components are parallel and perpendicular to the applied field^29, 30^. The component of the drift perpendicular to the electric field changes its sign with chirality of the spiral wave. A numerical study^31^ showed that depending on the parameter regime, the drift direction of the spiral could be either parallel (with small core) or antiparallel (with large core) to the dc electric field. The small core corresponds to highly excitable media, and the large core to weakly excitable media. The mechanism of the spiral drift by dc electric fields has been studied^25, 31–36^. However, most analytical studies are restricted to the weak or high excitability limit. By using the response function theory of spiral waves, the drift of spirals by dc electric fields was studied^28, 37, 38^. The theoretical results are quantitatively consistent with the numerical ones for both weak and high excitabilities. The theory was first proposed for the autonomous dynamics of scroll waves in the case of small curvatures and twists^39, 40^, and then extended to the drift of spiral waves in response to small perturbations^26^. Recently, an efficient numerical method of calculating response functions has been presented in an arbitrary model with differentiable right-hand sides^27, 41^. By using the response function theory, the drift laws for spiral waves on curved anisotropic surfaces were investigated. And an asymptotic theory that predicts the drift of spiral waves on general curved surfaces with the anisotropic diffusion has been developed^42^. The response function theory has also been used to study the change of the filament tension for scroll waves caused by a circularly polarized electric field^43^.

Spiral waves in the BZ reaction undergo a directional drift when the frequency of an ac electric field is twice that of the spiral frequency^44^. The direction of the spiral drift changes continuously between 0 and 2*π* when the initial rotation phase of the spiral varies from 0 to *π*. A kinematical model for spirals subjected to a strong ac electric field was proposed on a phenomenological basis^45^. This kinematical model has succeeded in capturing many aspects of the drift of spiral waves induced by strong ac electric fields. However, it has not been derived from the underlying reaction-diffusion equations. Thus, its parameters need to be adjusted, and cannot be obtained from the underlying reaction-diffusion equation. And the drift of spiral waves induced by weak ac electric fields has been studied from a theoretical point of view^25, 46^. But an analytical and quantitative explanation for this mechanism is needed.

Recently, a polarized electric field that possesses chirality was theoretically proposed^47^ and has been implemented in the BZ experiment^48^. It allows us to study the response of spiral waves to a chiral electric field. The drift behavior of spiral waves under the influence of a polarized electric field was investigated numerically. An analytical derivation which neglects the deformation of the spiral is consistent with the numerical results qualitatively^47^. However, an analytical derivation which takes account of the deformation for this mechanism is still lacking. Analytical results directly from the reaction diffusion equation are of importance, for a deeper understanding of the mechanism of the drift and a wider application of the spiral wave control.

In this paper, we derive the drift velocities of spiral waves due to weak ac and polarized electric fields using the response function theory, and compare the analytical results with the velocities obtained in direct numerical simulations for both weak and high excitabilities. The detailed forms of electric fields are shown in Table 1.

**Table 1:** Three forms of external electric fields. For ac electric fields, *ϕ*_*e*_ is the initial phase. For polarized electric fields, *ϕ*_*e*_ is the initial phase of *E*_*x*_, *ϕ*_*e*_ + *ϕ*_*xy*_ the initial phase of *E*_*y*_, and *ϕ*_*xy*_ the phase difference. The mode of the polarized electric field is characterized by *ϕ*_*xy*_. For examples, the polarized electric field is circularly polarized and rotates clockwise for *ϕ*_*xy*_ = 0.5*π*, or anticlockwise for *ϕ*_*xy*_ = 1.5*π*. See ref. 47 for more details. A small electric strength *E*_0_ = 0.005 is chosen throughout this paper.

dc electric fields	*E*_*x*_ = *E*_0_, *E*_*y*_ = 0
ac electric fields	*E*_*x*_ = *E*_0_ cos(*ω*_e_ *t* + *ϕ*_*e*_), *E*_*y*_ = 0
polarized electric fields	*E*_*x*_ = *E*_0_ cos(*ω*_e_ *t* + *ϕ*), *E*_*y*_ = *E*_0_ cos(*ω*_e_ *t* + *ϕ*_*e*_ + *ϕ*_*xy*_)

## Results

**Figure 1:**
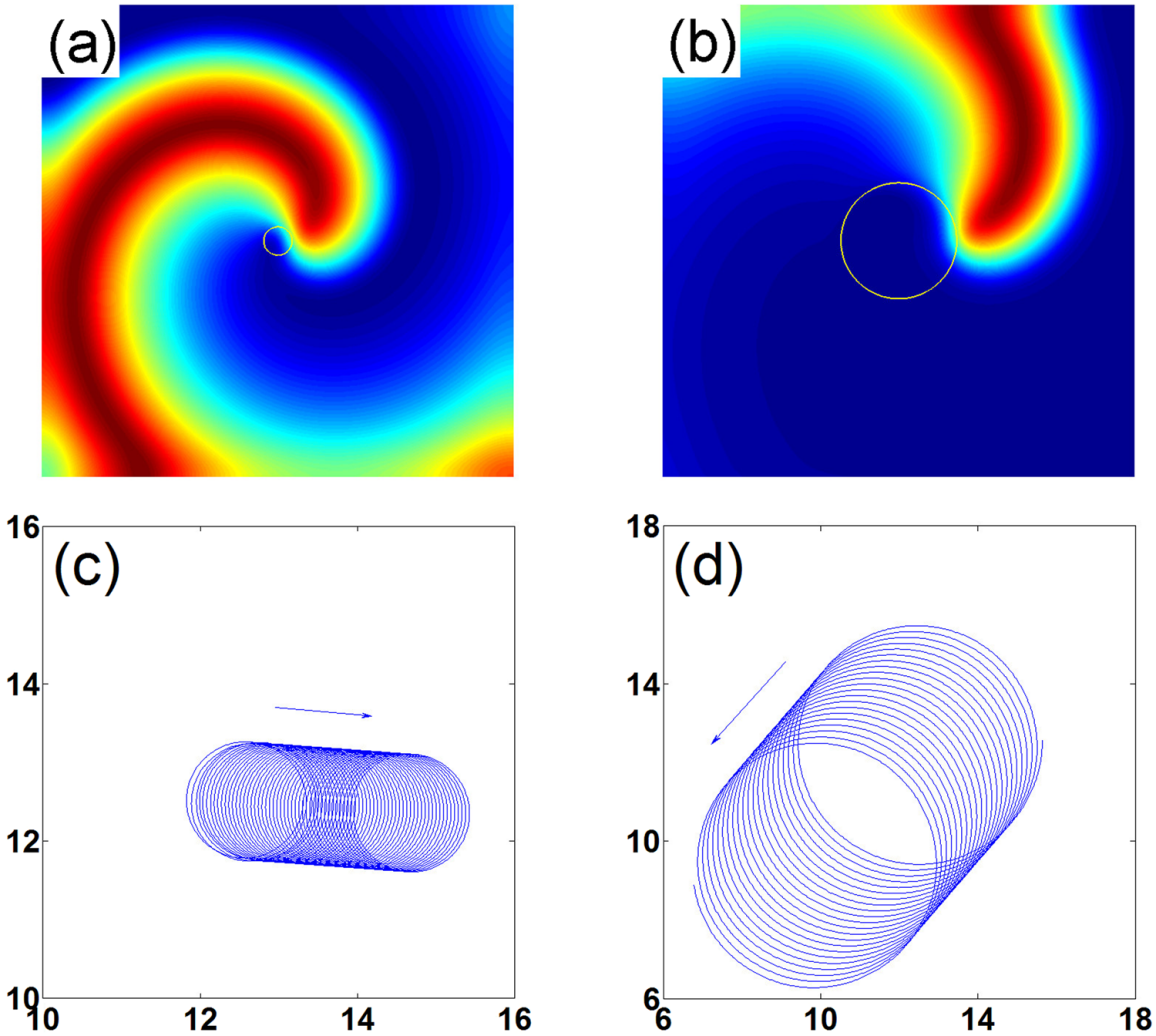
Top row: Tip trajectories of clockwise rotating spiral waves without electric field. Bottom row: Tip trajectories in the presence of a dc electric field. Left column: Highly excitable medium. Right column: Weakly excitable medium.

The theory of wave propagation in excitable media in the presence of an electric field $\overrightarrow{E}=({E}_{x},{E}_{y})$ can be described by reaction diffusion equations,

1 $${\partial }_{{\rm{t}}}{\bf{u}}={\bf{F}}({\bf{u}})+\mathop{{\bf{D}}}\limits^{{\boldsymbol{\frown }}{}}{\overrightarrow{\nabla }}^{{\rm{2}}}{\bf{u}}-\mathop{{\bf{M}}}\limits^{{\boldsymbol{\frown }}{}}\overrightarrow{E}\cdot \overrightarrow{\nabla }{\bf{u}}$$

where **u** = [*u*, *v*]^*T*^, ${\bf{F}}({\bf{u}})={[f(u,v),g(u,v)]}^{T}$, and $\mathop{{\bf{D}}}\limits^{{\boldsymbol{\frown }}{}}$ and $\mathop{{\bf{M}}}\limits^{{\boldsymbol{\frown }}{}}$ are constant matrices. In this paper, we use the FitzHugh-Nagumo kinetics^49, 50^, i.e., $f(u,v)=(u-{u}^{3}/3-v)/\varepsilon$ and $g(u,v)=\varepsilon (u+\beta -\gamma v)$. $\mathop{{\bf{D}}}\limits^{{\boldsymbol{\frown }}{}}$ and $\mathop{{\bf{M}}}\limits^{{\boldsymbol{\frown }}{}}$ are both set to be $$(\begin{array}{cc}1 & 0\\ 0 & 0\end{array})$$. Throughout this work, we choose a set of parameter values as *ε* = 0.22, *β* = 0.58, and *γ* = 0.8, such that the medium is highly excitable (see Fig. 1(a)). In the weakly excitable one, parameters are chosen as *ε* = 0.22, *β* = 0.78, and *γ* = 0.8 (see Fig. 1(b)). The spiral tip is defined as the intersections of two isolines of *u* = 0 and *v* = 0.

In the absence of electric fields, a rigidly rotating spiral wave solution to Eq. (1) is in the form of

2 $${\bf{U}}(\overrightarrow{r},t)={\bf{U}}(\rho (\overrightarrow{r}-\overrightarrow{R}),-\sigma {\vartheta }(\overrightarrow{r}-\overrightarrow{R})+\omega t+\sigma {\rm{\Phi }})$$

where $\overrightarrow{R}$ is the center of rotation, Φ is the initial rotation phase, *ω* > 0 is the angular frequency, and $\rho (\overrightarrow{r}-\overrightarrow{R})$ and ${\vartheta }(\overrightarrow{r}-\overrightarrow{R})$ are polar coordinates centered at $\overrightarrow{R}$. The spiral tip whose position vector is ${\overrightarrow{\zeta }}_{tip}(t)$ rotates in a circle centered at $\overrightarrow{R}$. Without loss of generality, we choose the angle between ${\overrightarrow{\zeta }}_{tip}(t=0)$ and the positive *x* axis as the initial rotation phase Φ, which is measured counterclockwise from the positive *x* axis. The rotation direction is determined by the chirality *σ*, i.e. *σ* = +1 for the counterclockwise rotating spiral and *σ* = −1 for the clockwise one^51, 52^.

### Derivation of the drift equations

In the following, without loss of generality, we only consider the drift of the clockwise rotating spiral, which can be easily extended to study the drift of the counterclockwise rotating spiral. It is convenient that the drift velocity of the spiral is analyzed in the system of reference of the spiral, i.e. the system of reference corotating with the spiral’s initial phase and angular velocity *ω* around the spiral’s center of rotation. In this system of reference, the polar angle is given by $\theta ={\vartheta }+\omega t-{\rm{\Phi }}$, with $\overrightarrow{R^{\prime} }=\mathop{0}\limits^{\rightharpoonup }$ and Φ′ = 0. According to the response function theory^26^, a small perturbation $-\mathop{{\bf{M}}}\limits^{{\boldsymbol{\frown }}{}}\overrightarrow{E}\cdot \overrightarrow{\nabla }{\bf{u}}$ acts on a rigidly rotating spiral by causing translational and rotational shifts. In particular, a solvability condition leads to the following equation for the drift velocity of the spiral:

3a $$\dot{R}(t)={e}^{i{\rm{\Phi }}}{e}^{-i\omega \tau }\langle {{\bf{W}}}^{(1)}({\rm{\Phi }}^{\prime} =0),-\mathop{{\bf{M}}}\limits^{{\boldsymbol{\frown }}{}}\overrightarrow{E}\cdot \overrightarrow{\nabla }{\bf{U}}({\rm{\Phi }}^{\prime} =0)\rangle $$

where $R(t)=X+iY$ is the complex coordinate of the instant spiral center, and $\dot{R}(t)$ is the drift velocity. The inner product 〈**w**, **v**〉 stands for the scalar product in the functional space in the system of reference of the spiral (Φ′ = 0)

$$\langle {\bf{w}},{\bf{v}}\rangle =\int {{\bf{w}}}^{+}(\rho ,\theta ){\bf{v}}(\rho ,\theta )\rho d\rho d\theta ,$$

and **W** ^(1)^ is one of the response functions of the spiral. Mathematically, it is the eigenfunction of the adjoint linearized operator corresponding to the critical eigenvalue −*iω*.

To study the directional drift of spirals, it is convenient to average the motion of the spiral over the rotation period. After the central moving average over the spiral wave rotation period, the drift velocity can be expressed as

3b $$\dot{R}={e}^{i{\rm{\Phi }}}{\int }_{{\rm{t}}-\pi /\omega }^{{\rm{t}}+\pi /\omega }\frac{\omega }{{\rm{2}}\pi }{e}^{-i\omega \tau }\langle {{\bf{W}}}^{(1)}({\rm{\Phi }}^{\prime} =0),-\mathop{{\bf{M}}}\limits^{{\boldsymbol{\frown }}{}}\overrightarrow{E}\cdot \overrightarrow{\nabla }{\bf{U}}({\rm{\Phi }}^{\prime} =0)\rangle {\rm{d}}\tau $$

$\dot{R}$ can also be written as $\dot{R}=|\dot{R}|{e}^{i{\rm{\Theta }}}$, where $|\dot{R}|$ is the drift speed, and Θ the drift direction. In Eq. (3b), $\mathop{{\bf{M}}}\limits^{{\boldsymbol{\frown }}{}}\overrightarrow{E}\cdot \overrightarrow{\nabla }{\bf{U}}=\mathop{{\bf{M}}}\limits^{{\boldsymbol{\frown }}{}}{E}_{x}{\partial }_{x}{\bf{U}}+\mathop{{\bf{M}}}\limits^{{\boldsymbol{\frown }}{}}{E}_{y}{\partial }_{y}{\bf{U}}$. It follows that

4a $$\begin{array}{rcl}{\partial }_{x}{\bf{U}} & = & {\partial }_{\rho }{\bf{U}}\,\cos \,\vartheta -{\rho }^{-1}{\partial }_{\vartheta }{\bf{U}}\,\sin \,\vartheta \\  & = & {\partial }_{\rho }{\bf{U}}\,\cos (\theta -\omega t+{\rm{\Phi }})-{\rho }^{-1}{\partial }_{\theta }{\bf{U}}\,\sin (\theta -\omega t+{\rm{\Phi }})\\  & = & -{{\bf{V}}}^{(1)}{e}^{i(\omega t-{\rm{\Phi }})}-{{\bf{V}}}^{(-1)}{e}^{-i(\omega t-{\rm{\Phi }})},\end{array}$$

4b $$\begin{array}{rcl}{\partial }_{y}{\bf{U}} & = & {\partial }_{\rho }{\bf{U}}\,\sin \,{\vartheta }-{\rho }^{-1}{\partial }_{\vartheta }{\bf{U}}\,\cos \,{\vartheta }\\  & = & {\partial }_{\rho }{\bf{U}}\,\sin (\theta -\omega t+{\rm{\Phi }})-{\rho }^{-1}{\partial }_{\theta }{\bf{U}}\,\cos (\theta -\omega t+{\rm{\Phi }})\\  & = & -i{{\bf{V}}}^{(1)}{e}^{i(\omega t-{\rm{\Phi }})}+i{{\bf{V}}}^{(-1)}{e}^{-i(\omega t-{\rm{\Phi }})},\end{array}$$

where

$$\begin{array}{rcl}{{\bf{V}}}^{(1)} & = & -\frac{1}{2}({\partial }_{\rho }{\bf{U}}-i{\rho }^{-1}{\partial }_{\theta }{\bf{U}}){e}^{-i\theta }\\ {{\bf{V}}}^{(-1)} & = & -\frac{1}{2}({\partial }_{\rho }{\bf{U}}+i{\rho }^{-1}{\partial }_{\theta }{\bf{U}}){e}^{i\theta }\end{array}$$

are two of the Goldstone modes of the spiral, i.e. the eigenfunctions of the linearized operator corresponding to the critical eigenvalues *iω* and −*iω*, respectively. Substituting Eq. (4) into Eq. (3b), we obtain finally

5 $$\begin{array}{c}\dot{R}={\int }_{{\rm{t}}-\pi /\omega }^{{\rm{t}}+\pi /\omega }\frac{\omega }{{\rm{2}}\pi }({E}_{x}+i{E}_{y})\langle {{\bf{W}}}^{(1)}({\rm{\Phi }}^{\prime} =0),\mathop{{\bf{M}}}\limits^{{\boldsymbol{\frown }}{}}{{\bf{V}}}^{(1)}({\rm{\Phi }}^{\prime} =0)\rangle {\rm{d}}\tau \\ \,\,\,\,\,+{e}^{i2\Phi }{\int }_{t-\pi /\omega }^{t+\pi /\omega }\frac{\omega }{{\rm{2}}\pi }{e}^{-i2\omega \tau }({E}_{x}-i{E}_{y})\langle {{\bf{W}}}^{(1)}({\rm{\Phi }}^{\prime} =0),\mathop{{\bf{M}}}\limits^{{\boldsymbol{\frown }}{}}{{\bf{V}}}^{(-1)}({\rm{\Phi }}^{\prime} =0)\rangle {\rm{d}}\tau .\end{array}$$

Thus a drift velocity formula in terms of the electric fields, the response functions, the Goldstone modes, and the initial rotation phase of the spiral is given.

For simplicity, we write the resultant as

6 $$\langle {{\bf{W}}}^{(1)}({\rm{\Phi }}^{\prime} =0),\mathop{{\bf{M}}}\limits^{{\boldsymbol{\frown }}{}}{{\bf{V}}}^{(1)}({\rm{\Phi }}^{\prime} =0)\rangle ={\mu }_{1}{e}^{i{\upsilon }_{1}},$$

7 $$\langle {{\bf{W}}}^{(1)}({\rm{\Phi }}^{\prime} =0),\mathop{{\bf{M}}}\limits^{{\boldsymbol{\frown }}{}}{{\bf{V}}}^{(-1)}({\rm{\Phi }}^{\prime} =0)\rangle ={\mu }_{2}{e}^{i{\upsilon }_{2}},$$

such that $|\dot{R}|$ and Θ can be explicitly written. In Table 2, we compute the drift coefficients *μ*_1_, *v*_1_, *μ*_2_, and *v*_2_ for both weak and high excitabilities using the open source software “DXSpiral”^41^.

**Table 2:** Values of the drift coefficients.

	*μ*_1_	*υ*_1_	*μ*_2_	*υ*_2_
high excitability	0.85671	6.2106	1.4897	0.60486
weak excitability	1.6826	3.9989	2.8027	5.5371

### DC electric fields

From the general drift velocity formula in Eq. (5), we first discuss the case of dc electric fields. When a dc electric field is applied, only the first component in Eq. (5) contributes to the directional drift of the spiral. And the drift velocity reads as

8 $$\dot{R}={E}_{0}\langle {{\bf{W}}}^{(1)}(\Phi ^{\prime} =0),\mathop{{\bf{M}}}\limits^{{\boldsymbol{\frown }}{}}{{\bf{V}}}^{(1)}(\Phi ^{\prime} =0)\rangle .$$

Substitution of Eq. (6) into Eq. (8) gives the drift speed and direction as

9 $$|\dot{R}|={\mu }_{1}{E}_{0},\quad {\rm{\Theta }}={\upsilon }_{1}.$$

From Table 2, one can calculate the theoretical predictions in two excitabilities as ${|\dot{R}|}_{weak}={\rm{0.008413}},$ ${{\rm{\Theta }}}_{weak}={\rm{3.999}}$ and ${|\dot{R}|}_{high}={\rm{0.004284}},\,{{\rm{\Theta }}}_{high}={\rm{6.211}}$, respectively. And from the direct numerical simulations (see Fig. 1(c) and (d)), we get ${|\dot{R}|}_{weak}={\rm{0.008415}},\,{{\rm{\Theta }}}_{weak}={\rm{4.004}}$ and ${|\dot{R}|}_{high}={\rm{0.004276}},\,{{\rm{\Theta }}}_{high}={\rm{6.209}}$ for the two excitabilities, respectively. Quantitative differences between theoretical predictions and numerical simulations are relatively small. The relative differences are less than 0.2% for both weak and high excitabilities. Equation (9) shows that both $|\dot{R}|$ and Θ are independent of the initial rotation phase Φ. This is also observed in numerical simulations. Note that the drift equations of spirals by dc electric fields in Eqs (8) and (9) are identical to the results obtained in refs. 28, 37, 38.

### AC electric fields

**Figure 2:**
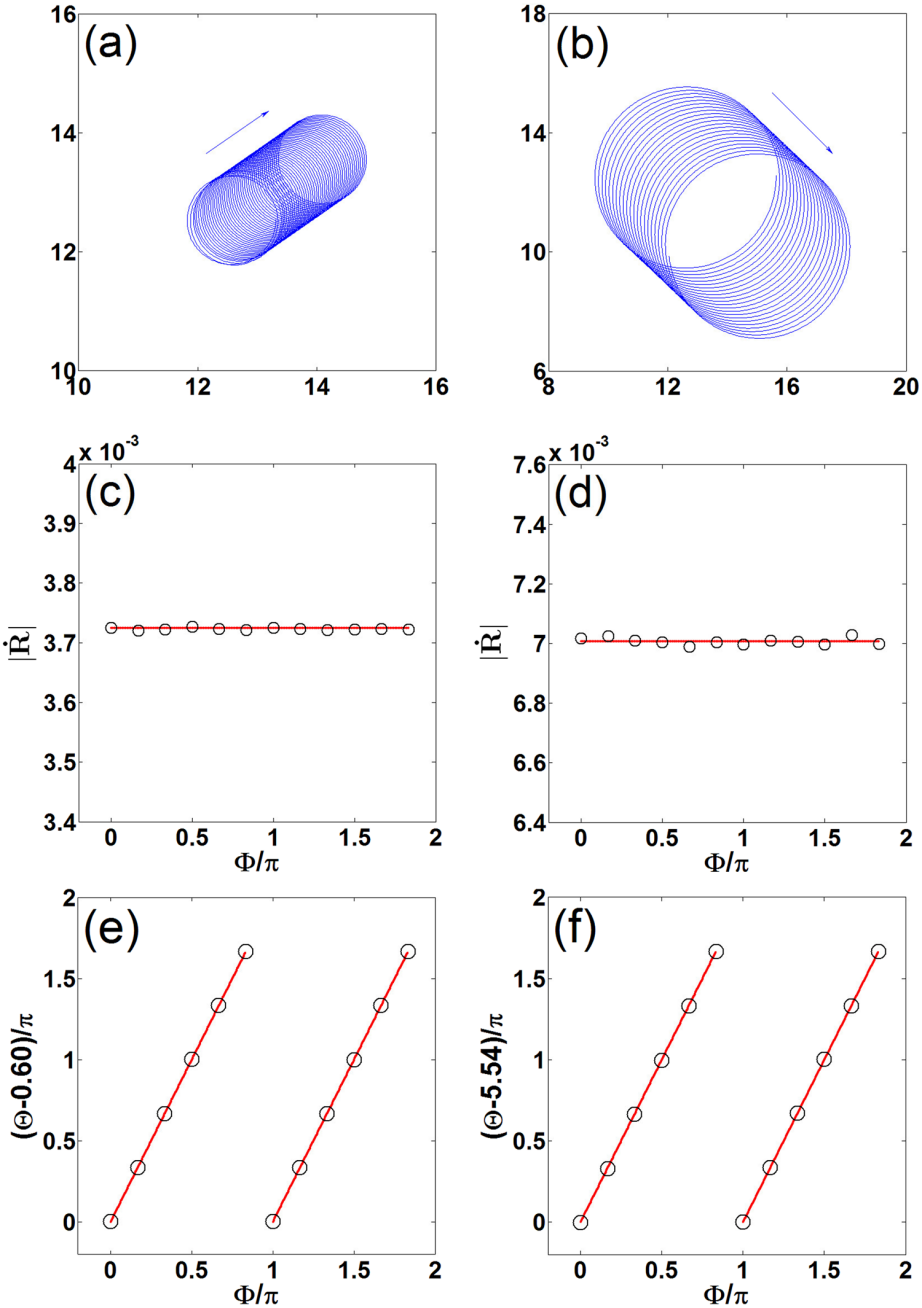
First row: Trajectories of spiral tips movement under the influence of an ac electric field with *ω*_*e*_ = 2*ω*, *ϕ*_*e*_ = 0, and Φ = 0. Second row: Drift speed as a function of Φ with *ϕ*_*e*_ = 0. Third row: Drift angle as a function of Φ with *ϕ*_*e*_ = 0. In the second and the third rows, the circles represent numerical simulations and the lines represent theoretical predictions. Left column: Highly excitable medium. Right column: Weakly excitable medium.

The spiral wave undergoes a directional drift in the presence of an ac electric field when *ω*_e_ = 2*ω* (see Fig. 2(a) and (b)). In this resonant case, the drift velocity in Eq. (5) becomes

10 $$\dot{R}=0.5{E}_{0}{e}^{i(2\Phi +{\varphi }_{e})}\langle {{\bf{W}}}^{(1)}(\Phi ^{\prime} =0),\mathop{{\bf{M}}}\limits^{{\boldsymbol{\frown }}{}}{{\bf{V}}}^{(-1)}(\Phi ^{\prime} =0)\rangle .$$

Using Eq. (7), one obtains

11 $$|\dot{R}|=0.5{\mu }_{2}{E}_{0},\quad {\rm{\Theta }}=2{\rm{\Phi }}+{\varphi }_{e}+{\upsilon }_{2}.$$

**Figure 3:**
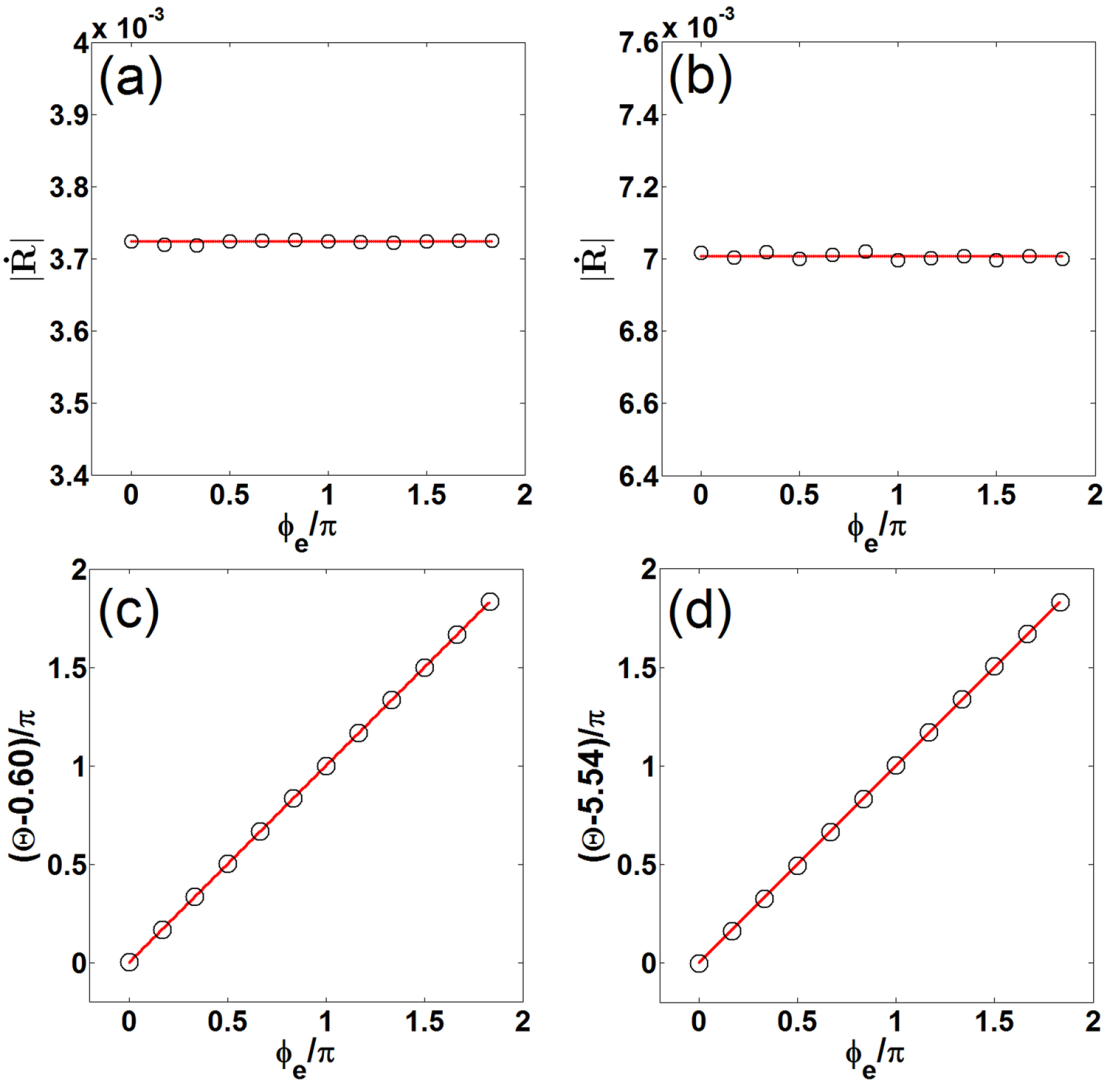
Drift velocity as a function of *ϕ*_*e*_ in the presence of an ac electric field with *ω*_*e*_ = 2*ω* and Φ = 0. Top row: Theoretical (lines) and numerical (circles) drift speed vs *ϕ*_*e*_. Bottom row: Theoretical (lines) and numerical (circles) drift angle vs *ϕ*_*e*_. Left column: Highly excitable medium. Right column: Weakly excitable medium.

From above equations, we can draw following conclusions about the resonant drift induced by ac electric fields, which are independent of the special models. Firstly, the drift speed $|\dot{R}|$ is independent of the initial rotation phase Φ of the spiral and the initial phase *ϕ*_*e*_ of the ac electric field, which are confirmed by direct numerical simulations in Figs 2(c),(d) and 3(a) and (b). The analytically obtained values $|\dot{R}|$ agree well with the ones obtained by simulations in both weakly and highly excitable media. Secondly, for the fixed *ϕ*_*e*_, the drift direction Θ changes continuously when the initial rotation phase Φ varies. And Θ is linear in Φ. The change of Θ is twice as much as that of Φ, i.e., ΔΘ = 2ΔΦ. Thus ΔΘ = 2π if ΔΦ = *π*. This means that the drift direction Θ keeps invariant when the change of Φ is *π*. The analytical values Θ are quantitatively consistent with the numerical simulations, as shown in Fig. 2(e) and (f). Thirdly, for the fixed Φ, the spiral drifts in different directions when we change *ϕ*_*e*_, but the change of Θ is equal to that of *ϕ*_*e*_, i.e., ΔΘ = Δ*ϕ*_*e*_. Numerical simulations are performed to demonstrate the quantitative agreement with the analytically obtained values Θ in both weakly and highly excitable media, as shown in Fig. 3(c) and (d). Note that the numerical and theoretical results of the spiral drift induced by strong ac electric fields in refs. 44 and 45 showed that $|\dot{R}|$ does depend on Φ, and Θ is not linear in Φ. The reason is clear. Since in refs. 44 and 45 *E*_0_ is equal to 2.0, but in our case *E*_0_ is 0.005. According to the response function theory, the analytical results obtained in this paper are valid for weak electric fields. On the other hand, the relation between Θ and *ϕ*_*e*_ was not studied in refs. 44 and 45. Thus, further laboratory works on the spiral drift induced by weak ac electric fields are expected. We note that spiral wave fronts may break above some electric field threshold^53^.

### Polarized electric fields

The general drift velocity formula in Eq. (5) can also be applied to the case of polarized electric fields. In the resonant case (*ω*_e_ = 2*ω*), the spiral drifts along a straight line, and the drift velocity reads as

12 $$\dot{R}=0.5{E}_{0}{{\rm{e}}}^{i(2\Phi +{\varphi }_{e})}(1-i{e}^{i{\varphi }_{xy}})\langle {{\bf{W}}}^{(1)}({\rm{\Phi }}^{\prime} =0),\mathop{{\bf{M}}}\limits^{{\boldsymbol{\frown }}{}}{{\bf{V}}}^{(-1)}({\rm{\Phi }}^{\prime} =0)\rangle .$$

Substituting Eq. (7) into Eq. (12), one can obtain

13 $$\begin{array}{rcl}|\dot{R}| & = & 0.5{\mu }_{2}{E}_{0}\sqrt{2(1+\,\sin \,{\varphi }_{xy})},\\ {\rm{\Theta }} & = & 2{\rm{\Phi }}+{\varphi }_{e}+0.5{\varphi }_{xy}+{\upsilon }_{2}+0.75\pi ,\quad {\varphi }_{xy}-1.5\pi \in (0,2\pi ).\end{array}$$

**Figure 4:**
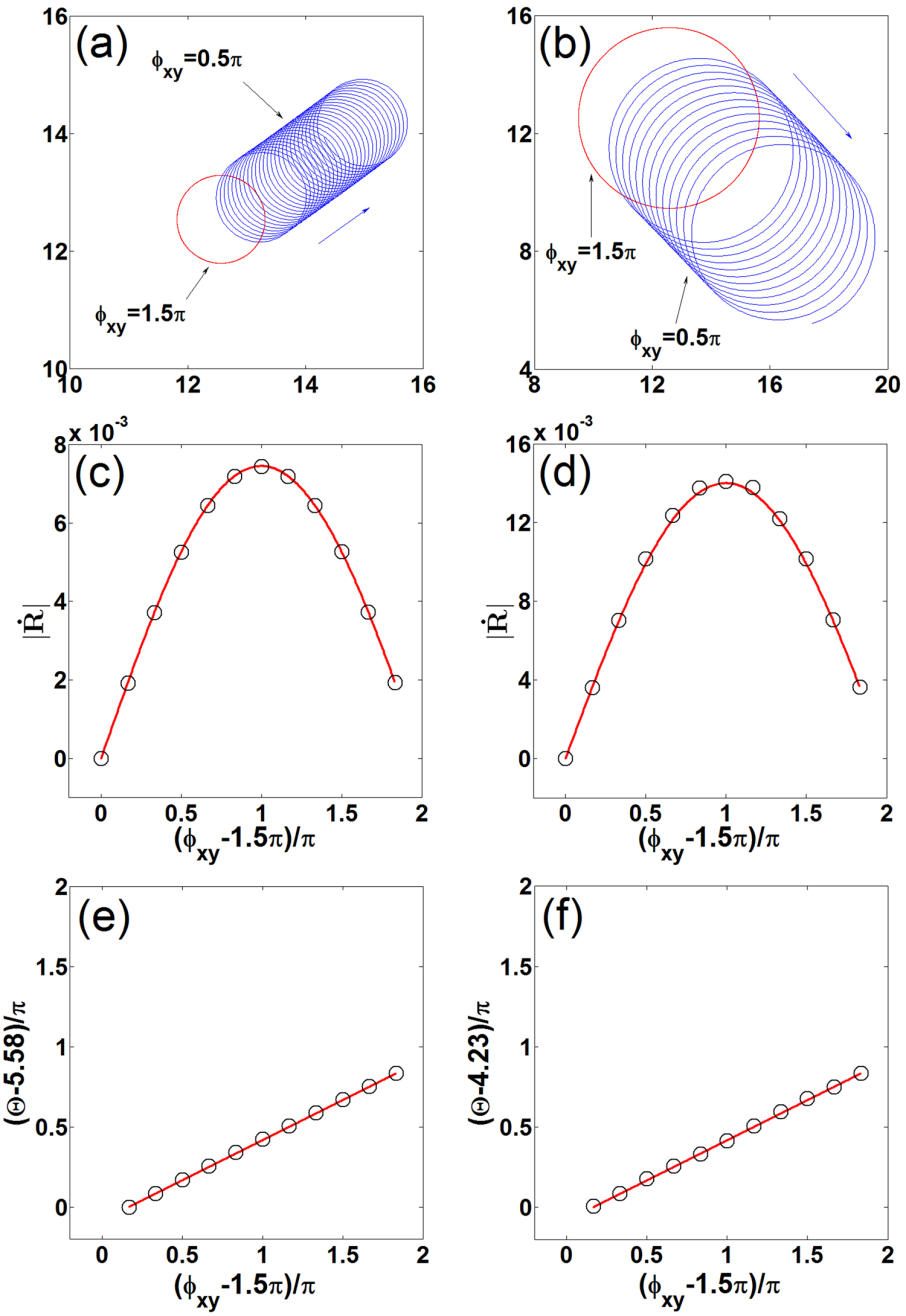
First row: Drifting behaviors of spirals under the influence of a clockwise (*ϕ*_*xy*_ = 0.5*π*) and a counterclockwise (*ϕ*_*xy*_ = 1.5*π*) circularly polarized electric fields with *ω*_*e*_ = 2*ω*, *ϕ*_*e*_ = 0, and Φ = 0. Second row: Dependence of theoretical (lines) and numerical (circles) drift speed on *ϕ*_*xy*_ with *ϕ*_*e*_ = 0 and Φ = 0. Third row: Dependence of theoretical (lines) and numerical (circles) drift angle on *ϕ*_*xy*_ with *ϕ*_*e*_ = 0 and Φ = 0. When the drift speed is 0 (*ϕ*_*xy*_ = 1.5*π*), the drift angle cannot be defined. Left column: Highly excitable medium. Right column: Weakly excitable medium.

A significant feature predicted in Eq. (13) is that when the electric field is circularly polarized and its rotation follows that of the spiral (*ϕ*_*xy*_ = 0.5*π*), the drift speed $|\dot{R}|$ reaches its maximal value. On the contrary, opposite rotation between the spiral and electric field (*ϕ*_*xy*_ = 1.5*π*) locks the spiral. This prediction is confirmed by numerical simulations, and the analytically obtained values $|\dot{R}|$ are quantitatively consistent with the ones obtained by simulations, as shown in Fig. 4(a–d). And the drift direction Θ changes continuously when Φ or *ϕ*_*e*_ varies. Their relations are ΔΘ = 2ΔΦ and ΔΘ = Δ*ϕ*_*e*_, which are the same results as in ac electric fields. Moreover, the spirals drift in different directions when we change the phase difference *ϕ*_*xy*_, and the change of the drift direction is half as much as that of the phase difference, i.e., ΔΘ = 0.5Δ*ϕ*_*xy*_. This unexpected prediction is also confirmed by direct numerical simulations, and the analytical values Θ agree well with the numerical ones, as shown in Fig. 4(e,f).

In conclusion, we have studied the drift of spiral waves induced by weak electric fields analytically, since analytical results are of crucial importance for a deeper and more comprehensive understanding to the mechanism of the spiral wave drift. Using the response function theory, we propose a theory of the spiral wave drift due to weak ac and polarized electric fields. Explicit equations for the spiral wave drift speed and direction in terms of Φ, *E*_0_, *ϕ*_*e*_, and *ϕ*_*xy*_ are obtained directly from the reaction diffusion equations, which are independent of the special models and should be of general significance. These analytical results are quantitatively confirmed by numerical simulations in both weakly and highly excitable media. Although ac electric fields^44^ and polarized electric fields^48^ have been realized in the BZ system, laboratory works on the spiral drift induced by weak ac and polarized electric fields have not been investigated. We hope that the theoretical results in Eqs (11) and (13) can be verified in experiments.

## Methods

In direct numerical simulations of the FitzHugh-Nagumo model in Eq. (1), we use an explicit Euler method and no-flux boundary conditions with the space step at Δ*x* = Δ*y* = 0.05 and time step at Δ*t* = 0.0005 for the grids 500 × 500 in Cartesian coordinate system.

We compute **V** ^(1)^(Φ′ = 0), **V** ^(−1)^(Φ′ = 0), and **W** ^(1)^(Φ′ = 0) using the open source software “DXSpiral”^41^. Since “DXSpiral” is dealing with spiral waves on a polar grid in a disk, to get a similar precision with Δ*x* = 0.05 in direct numerical simulations, we use a disk of radius at 12.5 with 250 radial and 124 circumferential grid cells. Note that in the computation, the initial rotation phase of the spiral in the system of reference of the spiral should be set to be 0, i.e. Φ′ = 0. In our computations, we find that $\langle {{\bf{W}}}^{(1)}({\rm{\Phi }}^{\prime} ),\mathop{{\bf{M}}}\limits^{{\boldsymbol{\frown }}{}}{{\bf{V}}}^{(-1)}({\rm{\Phi }}^{\prime} )\rangle$ is dependent on Φ′, but $\langle {{\bf{W}}}^{(1)}({\rm{\Phi }}^{\prime} ),\mathop{{\bf{M}}}\limits^{{\boldsymbol{\frown }}{}}{{\bf{V}}}^{(1)}({\rm{\Phi }}^{\prime} )\rangle$ is not. This is related to the fact that the drift velocity of the spiral induced by the dc electric field does not depend on the initial rotation phase of the spiral, while the drift velocity induced by the ac electric field depends on the initial rotation phase.
